# Surgical Excerpts

**Published:** 1858-01

**Authors:** 


*Surgical Excerpts.—Calculus.*—Dr. Broenner relates (Wurtzburg Verhandlunger, 1856) a case of urinary calculus in an infant of one year, evacuated spontaneously. This concretion was composed of urates, and was six millemetres in diameter. Its passage through the urethra was accompanied by violent symptoms and almost complete retention of urine.—*Ib.*

